# Molecular imaging biomarkers for cell-based immunotherapies

**DOI:** 10.1186/s12967-017-1240-6

**Published:** 2017-06-19

**Authors:** Mohammad Haris, Puneet Bagga, Hari Hariharan, Bevin McGettigan-Croce, Laura A. Johnson, Ravinder Reddy

**Affiliations:** 1 Center for Magnetic Resonance and Optical Imaging (CMROI), Department of Radiology, Perelman School of Medicine at the University of Pennsylvania, B1 Stellar-Chance Laboratories, 422 Curie Boulevard, Philadelphia, PA 19104-6100 USA; 2 Translational Medicine, Sidra Medical and Research Center, Doha, Qatar; 3 Center for Cellular Immunotherapies, Perelman School of Medicine at the University of Pennsylvania, Philadelphia, USA; 4 Department of Pathology and Laboratory Medicine, Perelman School of Medicine at the University of Pennsylvania, Philadelphia, USA

## Abstract

While many decades of scientific research studies have gone into harnessing the power of the immune system to fight cancer, only recently have cancer immunotherapeutic approaches begun to show robust clinical responses in patients with a variety of cancers. These treatments are adding to the current arsenal of cancer treatments; surgery, radiation and chemotherapy, and increasing the therapeutic options for cancer patients. Despite these advances, issues associated with these therapies include that not all patients respond to these therapies, and some patients who respond experience varying degrees of toxicities. One of the major issues affecting immunotherapy is the inability to evaluate trafficking of activated T-cells into sites of tumor. The current diagnostic imaging based on conventional anatomic imaging, which is the mainstay to monitor response to cytotoxic chemotherapy or radiation, is not adequate to assess initial response to immunotherapy or disease evolution. Patients’ prognosis by histological analysis has limited use in regards to immunotherapy. Thus, there is a crucial need for noninvasive biomarkers for screening patients that show long term response to therapy. Here, we provide a brief account of emerging molecular magnetic resonance imaging biomarkers that have potential to exploit the metabolism and metabolic products of activated T cells.

## How cancer cheats the immune system

Effective anti-cancer treatments are largely affected by the cross-talk between cancer and the patient’s immune system. Studies have demonstrated that tumors evade the host immune response via a number of mechanisms [1–3]. In down regulating the major histocompatibility complex I, cancer cells make the detection of the antigens on their surface by the immune system less effective [4, 5]. Further, they produce immunosuppressive cytokines, such as (TGF-β) and interleukin-10 (IL-10) that down regulate the cytotoxic immune cells and shift the immune response towards a suppressive phenotype [4, 6]. Finally, they upregulate surface proteins such as programmed cell death-ligand 1 (PD-L1), an important protein of normal cells in preventing autoimmune phenomena [6–8]. When the PD-1 receptor on cytotoxic T cells interacts with PD-L1, the T-cells become anergic and do not destroy them.

## Immune system for cancer therapy

In the past few years, one of the most exciting advances in the treatment of tumors is boosting the body’s immune response against cancer [9]. There are different approaches to boost or restore immune function against cancer, which are broadly classified into four categories: immune check point blockade [8, 10], adoptive T-cell therapy [11, 12], exogenous cytokines [13, 14] and therapeutic vaccines [15, 16].

### Check point blockade

Recent advances have demonstrated that blockade of immune checkpoints is one of the most promising approaches for activating therapeutic antitumor immunity [8]. Immune checkpoints are the receptor-ligand pairs on the cell surface that are involved in regulating T-cell activation.

It is now established that tumors utilize certain immune-checkpoint pathways as a mechanism of immune resistance against T cells that are specific for tumor antigens. Since many of the immune checkpoints involve ligand-receptor interactions, they can be readily blocked by antibodies or modulated by recombinant forms of ligands or receptors. Immunotherapeutics based on antibodies of cytotoxic T-lymphocyte-associated antigen 4 (CTLA4) [17, 18] and programmed cell death protein-1 (PDCD1/PD1) are showing promising results of antitumor immunity [19, 20]. In fact, the immunomodulatory monoclonal antibody of CTLA4, Ipilimumab, is the first Food and Drug Administration (FDA) approved immunotherapeutic agent for treating cancer [10, 21]. More recently, Nivolumab and Pembrolizumab, humanized IgG4 antibodies, which block PD-1 and inhibits its interaction with PD-L1 and PD-L2 have also been approved as immunotherapeutic agents for treatment of cancer by the US FDA [22–24].

### Adoptive T-cell therapies

Adoptive T-cell therapies include expanded autologous T cells and T cells with engineered T-cell receptors (TCRs) and chimeric antigen receptors (CARs) [25, 26]. Specifically, tumor-infiltrating lymphocytes (TILs) are isolated from tumor biopsies and expanded before being reinfused into the patient, based on the premise that these TILs are tumor cell specific. The most effective T-cell therapies explored in clinical trials currently focus on leukemia, but are also used to treat patients with solid tumors.

### Cytokines

Cytokines play important roles in the body’s normal immune responses and also in the immune system’s ability to respond to cancer. Interferons and interleukins are two main types of cytokines used to treat cancer [13, 14]. These cytokines bind to their receptors on T cells, and stimulate the activation and proliferation of T cells and downstream production of more cytokines [13, 14].

### Treatment vaccines

These vaccines stimulate an active immune response against tumor by eliciting adaptive immunity through the patient’s own immune system. After injection of peptide or protein vaccines, the body’s antigen presenting cells (APCs) process vaccines as antigenic fragments to be recognized and stimulate the patient’s naïve T cells, which in turn may stimulate an endogenous immune response against cancer [15, 16].

### Problems

While these immunotherapy methods provide tremendous hope for patients, they also present significant challenges. Treatment with immunotherapies is showing new patterns of treatment response and side effects. Specifically, after immunotherapy the response can be manifested different ways: (1) a decrease in size of known tumors without the presence of new tumor after completion of treatment, (2) clinically stable disease after completion of treatment and significantly delayed decrease in tumor size, (3) new or enlarging tumors observed soon after completion of treatment, which may not reflect disease progression, preceding a later decrease in tumor burden (4) autoimmune-mediated toxic effects that could be mistaken for metastatic disease or misdiagnosed as a non-treatment-related process and delay appropriate clinical management [27].

Currently, there are no robust biomarkers to identify the patients who will most likely benefit from these treatments. In the absence of a predictive biomarker, many patients may receive these expensive treatments without any benefit. These unconventional treatment response patterns and the wide range of autoimmune toxic effects make it rather challenging to monitor the effects of immunotherapies using Response Evaluation Criteria in Solid Tumors (RECIST) [28] criteria, which are based on the conventional anatomical imaging by computed tomography and magnetic resonance imaging (MRI) [29]. Hence, there is need for robust technology, which not only characterizes the immune microenvironment of tumors but also screen for patients who can potentially respond to immunotherapies. Imaging methods targeting T cell metabolism have the potential for providing molecular imaging biomarkers to assess immunotherapy response. To develop molecular imaging biomarkers, understanding the T cell metabolism and its changes upon activation are crucial.

## Regulation of T cell metabolism

**Fig. 1:**
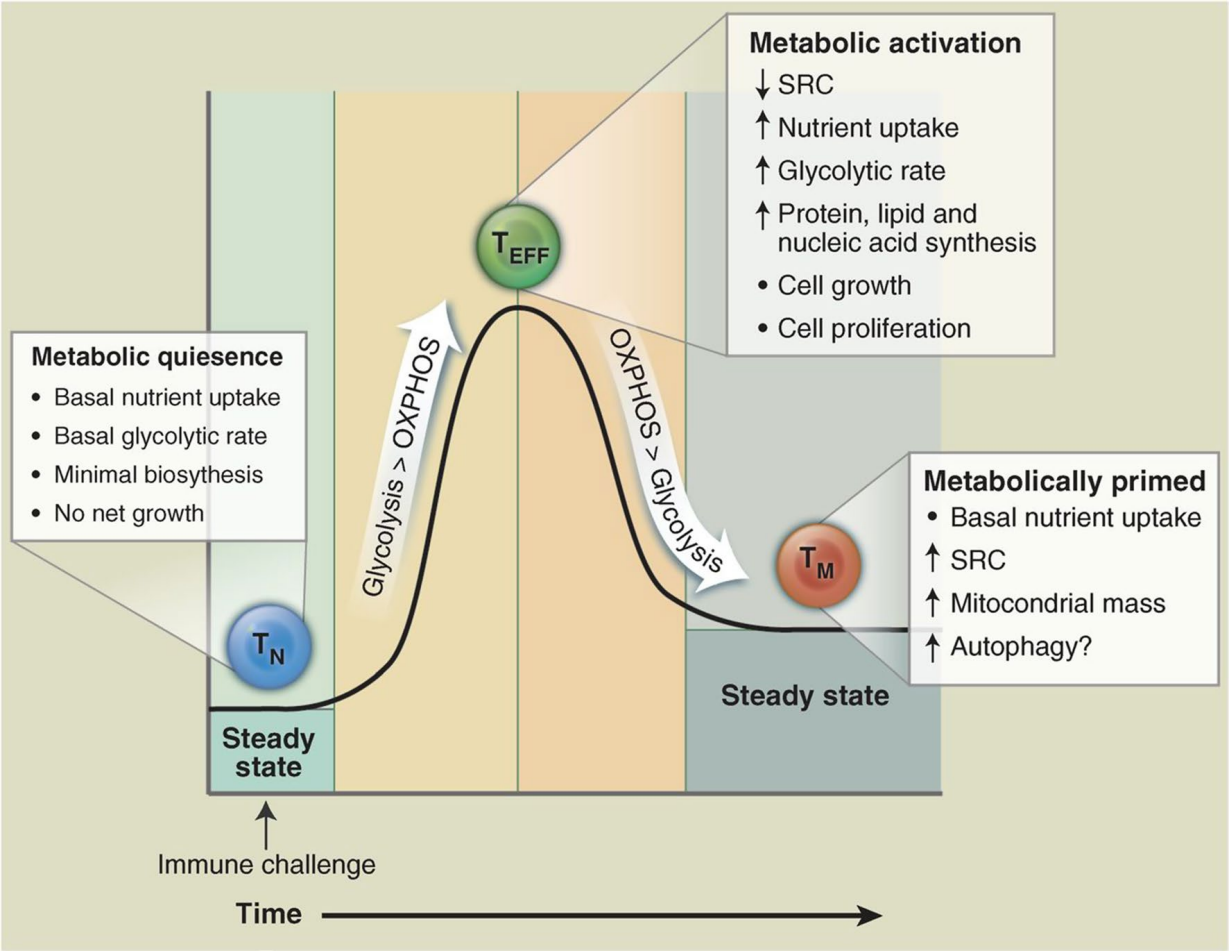
Showing the diagrammatic representation of metabolic switch in different stages of T cells. Naïve T cells (T_N_) are predominantly depending on the OXPHOS for the energy needs. The proliferative T cells (T_EFF_) shifts to aerobic glycolysis to support their high energy demand. The memory T cells (T_M_) use OXPHOS pathway for their metabolic requirement [32]. Reprinted with permission from AAAS

Like all non-proliferating cells, naïve T cells (T cells that have not yet encountered antigen) adopt a basal level of nutrient uptake and primarily use oxidative phosphorylation (OXPHOS) for adenosine triphosphate (ATP) production. When the T cells encounter antigen (on tumors) they become activated and respond by extensive proliferation and differentiation into effector T cells (T_EFF_), which identify and eradicate pathogenic threats to the host systems. In the activated state, the T_EFF_ cells switch to anabolic growth and biomass accumulation to generate daughter cells that increases the demand for ATP. To support their high energy demand, activated T cells shifts to aerobic glycolysis, which involves conversion of glucose derived pyruvate to lactate even in the presence of oxygen for glucose oxidation-also known as Warburg effect. Although both CD4+ and CD8+ T_EFF_ cells still engage OXPHOS, they predominantly employ aerobic glycolysis [30, 31]. After clearing the pathogens, most T_EFF_ cells die, and a small population of long-lived antigen-specific memory T cells (T_M_) are left behind. Like naïve cells, the T_M_ cells engage OXPHOS and maintain lower rates of nutrient uptake and biosynthesis when compared to T_EFF_ cells (Fig. 1) [32].

**Fig. 2:**
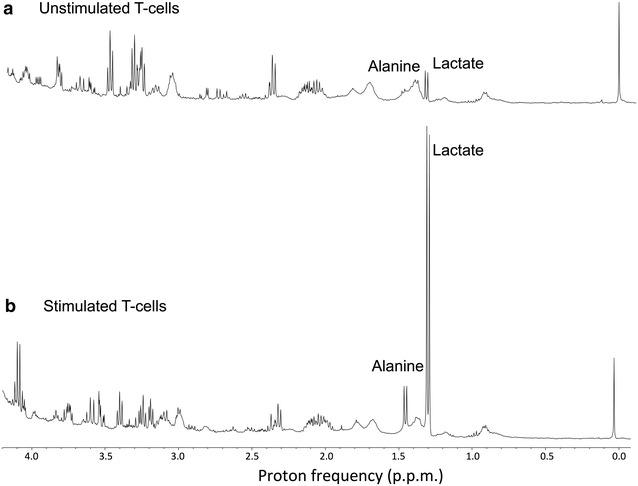
^1^H NMR spectra from cultured T cells obtained at 9.4 T. **a** Spectrum from previously non-stimulated resting T-cells shows lactate and other intra cellular metabolites, and very little alanine. **b** Spectrum from stimulated T cells with CD3/CD28 obtained under identical conditions of the spectra in **a**, which shows substantially (over 12-fold increase) higher lactate and alanine (3-fold increase) compared to that from non-stimulated resting T-cells (unpublished results from author’s laboratory)

The above discussion implies that in the activated form T cells (T_EFF_) predominantly engage aerobic glycolysis, just like proliferating cancer cells. It is well known that predominant product of aerobic glycolysis is lactic acid generation. So, cancer cells in a tissue, as a result of aerobic glycolysis, produce lactate and maintain an acidic environment. We hypothesize that upon activation, the T_EFF_ cells will also go through the glycolysis and produce significant amount of lactic acid and increases the overall lactate level in the cancer tissue. This increased lactate level may serve as a biomarker for T cell activation and engaging the cancer cells. In addition to lactate increase, T_EFF_ cells will also show significant increase in Alanine (Fig. 2). Upon successful immunotherapy, both cancer cells as well as the T_EFF_ cell decrease substantially and overall lactate levels will also decrease accordingly and normalize to basal levels over a period of time. So, rapid changes in glycolysis, amino acids and proteins in CAR T_EFF_ cells, lactate production and pH changes potentially serve as molecular biomarkers for therapeutic response and disease progression.

Among the molecular imaging techniques, ^18^F-fluorodeoxyglucose (^18^F-FDG) positron emission tomography (PET) imaging of cancer is the most studied modality in oncologic nuclear imaging [33]. It is utilized primarily to assess tumor glycolysis among other things. However, primary challenges with ^18^F-FDG-PET include its inability to differentiate between cancer and infectious or inflammatory processes. Specifically, this becomes major shortcoming when evaluating response to therapy amid immune-related adverse events after treatment with immunotherapy agents. While ^18^F-fluorothymidine, a marker of cell proliferation, which was developed to identify viable tumor, it is beset by lower signal to background ratio compared with ^18^F-FDG-PET and uptake in background structures, accumulation in sites of infection and inflammation can limit detection and quantification of tumor activity [34, 35]. Furthermore, frequent imaging with ^18^F-FDG-PET is not feasible as it involves radiation.

MRI can provide high resolution anatomical imaging along with an array of functional measures: tumor perfusion, diffusion, cell membrane permeability via contrast enhanced MRI, immune cell tracking using magnetic iron oxide particles, etc. However, for the reasons mentioned above, these measures are not adequate for assessing response to immunotherapy.

## Chemical exchange saturation transfer (CEST) MRI of T cell metabolites

One of the challenges in the diagnosis of response to immunotherapy is distinguishing between new tumor and inflammation or edema. CEST methods potentially address this issue. Recent developments in CEST methods show that it is feasible to image metabolites such as glutamate [36, 37], creatine [38], glucose [39], glycogen [40], myoinositol [41], lactate [42] and glycosaminoglycans [43]. In the activated state, the T cells go through the glycolysis to support rapid energy required for biosynthesis of daughter cells. This leads to accumulation of metabolites such as lactate, creatine, choline, glutamate and alanine in T_EFF_ cells. Monitoring changes in these metabolites level pre- and post immune therapy has potential to assess the relative changes in the T_EFF_ cell density.

### CEST MRI of lactate: a biomarker for immunotherapy

Currently there are two major methods employed in measuring lactate in vivo. One is traditional magnetic resonance spectroscopy (MRS; both ^1^H and ^13^C) [44–46], which has been used to measure both static lactate levels and dynamic changes. However, these are limited by inadequate sensitivity and spatial resolution. The other method involves infusion of dynamic nuclear polarized (DNP)^13^C-labeled pyruvate, which provides greater than 10,000-fold signal enhancement compared to conventional MRS [47–49]. Despite its high sensitivity, this method only probes fast kinetics (<1 min) of lactate turnover from ^13^C-labeled pyruvate and it requires special equipment and complex modeling for data analysis.

**Fig. 3:**
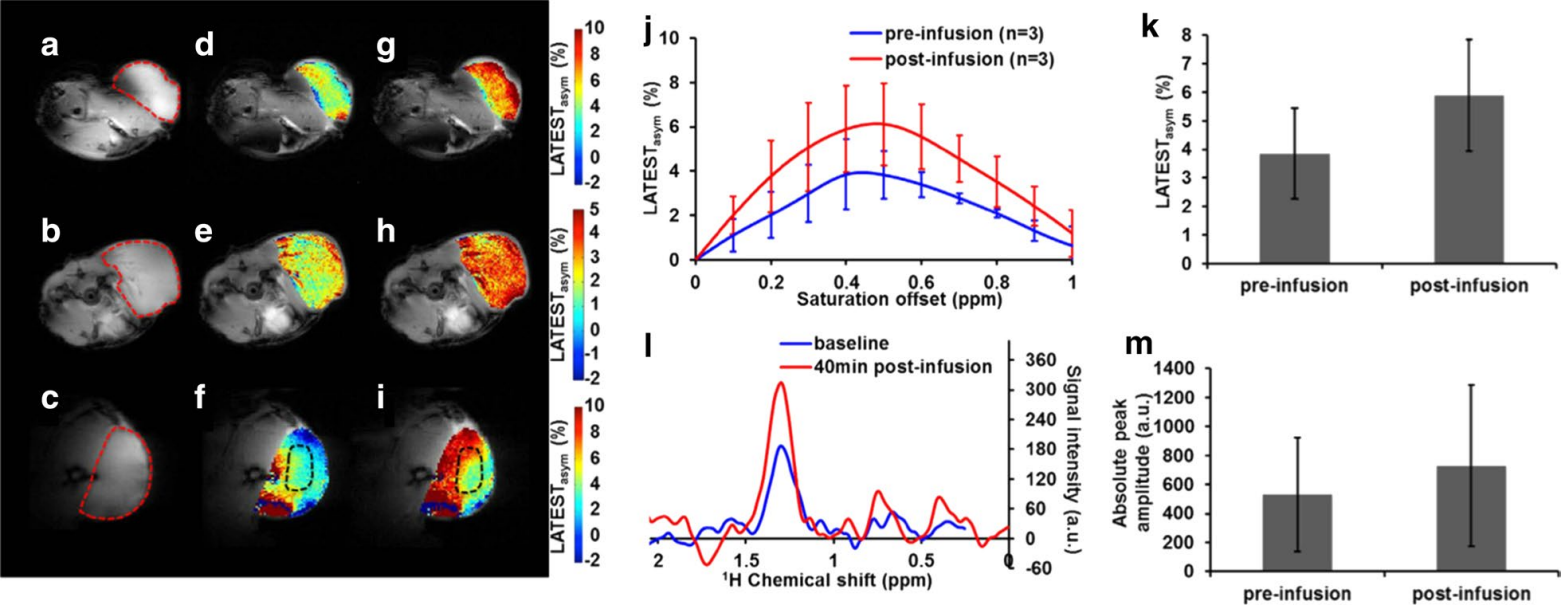
LATEST from lymphoma tumors [42]. **a**–**c** Anatomical image from three animals, with flank tumor region indicated by *dotted red line*, and the LATEST maps (**d**–**f**) pre-infusion and (**g**–**i**) post-infusion with (**j**) corresponding asymmetry plots (asymmetry from Animal 3 in the third row is taken from region indicated in *dotted black line*), (**k**) LATEST change at 0.4 ppm from three animals pre- and post-infusion, (**l**) representative SEL-MQC^1^H-MRS pre- and 40 min post-infusion from flank tumor showing (**m**) increase in lactate peak amplitude from three animals (~40%) from spectroscopy. Reproduced with permission

Recently, MRI method based on lactate CEST (LATEST) [42] to image lactate was described. LATEST method utilizes standard proton MRI and requires neither ^13^C labeled pyruvate nor DNP polarization. The feasibility of measuring LATEST in vivo was demonstrated in a lymphoma tumor model (Fig. 3), and in human skeletal muscle [42]. Dynamic changes in LATEST are reported in tumors pre- and post-infusion of pyruvate, and in exercising human skeletal muscle [42]. LATEST measurements are compared to lactate measured with multiple quantum filtered proton MRS [42]. LATEST provides over two orders of higher sensitivity compared to the ^1^H MRS based lactate detection methods.

It was reported that lactic acid produced by the tumor cells blunts the tumor immunosurveillance by T and natural killer cells. This implies that the higher levels of lactate in tumor cells may adversely affect the immunotherapy and basal levels of lactate itself might give a clue regarding the response to immunotherapy [50]. In the context of immunotherapy, pre-therapy LATEST images provide the basal levels of lactate in tumor regions which are largely glycolytic and produce more lactate. Immediately, post-therapy (12–24 h), if the immune cells have identified receptors on the tumors cell surface and get activated then the T_EFF_ cells switch their metabolism to glycolysis and begin to proliferate rapidly and dump lots of lactate into the tumor microenvironment (at this time tumor cells may be still be producing lactate, although with a different/slower rate). This rapid increase in lactate can be measured using LATEST. In addition to this rapid increase in LATEST, T cells activation may also lead to side effects associated with autoimmunity. As the T cell rapidly proliferates, it may lead to an increase in the size of the T cells mass in the tumor region, which is often mistaken for tumor growth. This elevation in the lactate levels remains until the T cells completely destroy the tumor cells and then levels begin to drop, as the T_EFF_ cells die and convert to T_M_ cells, to basal values.

On the other hand, if the immune cells do not get activated then their metabolism remains OXPHOS and there would not be any change in the lactate levels due to immune cells and as the tumor cells are continuously proliferating, lactate levels and tumor size increase gradually. So the kinetics of the lactate measured shed light on the therapeutic efficacy.

The slopes of the lactate concentration vs. time curves, especially hours after the treatment, will serve as a measure of the response. Response to therapy is expected to produce a steeper slope in the curve than no response.

### CEST MRI of glutamate, alanine and creatine

Higher concentration of glutamate, alanine and creatine during the T cell proliferation in response to immunotherapy can also be monitored using CEST. Studies have shown that the changes in these metabolites level in cancer tissue can be monitored non-invasively through CEST. Different CEST based approaches (GluCEST, glutamate; AlaCEST, alanine; CrCEST, creatine) have been developed to image these metabolites in vivo. In addition, another CEST method, amide proton transfer (APT), which primarily depends on the mobile protein content, has been shown to be useful in discriminating between tumor regrowth and radiation necrosis [51]. It has been shown that the glutamate released by the dendritic cells mediate**s** the T cell activation/proliferation [52]. Higher expression of glutamate metabotropic receptor on activated T cells further confirms the role of the glutamate in T cells mediated immunity [52]. The increase in alanine concentration in an in vitro stimulated T cell line (Fig. 2) suggests that the activation of T cells result in more alanine synthesis. Changes in the in vivo glutamate, alanine and creatine level post-immunotherapy as measured by CEST may also serve as potential biomarkers to evaluate the treatment response.

## Conclusion

Overall, immediately after the immunotherapy administration, rapid increase in lactate (derived via LATEST) and glutamate, alanine, and creatine from tumor regions is indicative of response to immunotherapy. Successful therapy eventually will be manifested in favorable clinical symptoms as well as low values of LATEST and CrCEST (or GluCEST/AlaCEST) compared to post-treatment. If during post treatment phase inflammation occurs or edema builds up then neither LATEST nor CrCEST (or GluCEST/AlaCEST) would increase. Thus compared to pre-treatment, unchanged or small changes in LATEST and CrCEST (or GluCEST/AlaCEST) values, in tumor region post treatment, may point to unresponsiveness. The CEST MRI methods potentially provide an early biomarker to monitor the immunotherapy response in vivo and to evaluate the patients who will respond to immunotherapy.

## Abbreviations

MRI: magnetic resonance imaging
PD-L1: programmed cell death-ligand 1
CTLA4: cytotoxic T-lymphocyte-associated antigen 4
PDCD1/PD1: programmed cell death protein-1
FDA: Food and Drug Administration
TCRs: engineered T cell receptors
CARs: chimeric antigen receptors
TILs: tumor-infiltrating lymphocytes
APCs: antigen presenting cells
RECIST: Response Evaluation Criteria in Solid Tumors
ATP: adenosine triphosphate
TEFF: effector T cells
OXPHOS: oxidative phosphorylation
TM: memory T cells
18F-FDG: 18F-fluorodeoxyglucose
PET: positron emission tomography
CEST: chemical exchange saturation transfer
DNP: dynamic nuclear polarization
MRS: magnetic resonance spectroscopy
LATEST: lactate CEST
APT: amide proton transfer
